# Analysis of Current Situation, Demand and Development Trend of Casting Grinding Technology

**DOI:** 10.3390/mi13101577

**Published:** 2022-09-22

**Authors:** Haigang Liang, Jinwei Qiao

**Affiliations:** 1 School of Mechanicaland Automotive Engineering, Qilu University of Technology (Shandong Academy of Sciences), Jinan 250353, China; 2 Shandong Institute of Mechanical Design and Research, Jinan 250353, China

**Keywords:** robot grinding, visual control, casting post-processing

## Abstract

Although grinding is essential in the production of castings, the casting grinding process in manufacturing is complicated and there are many difficulties, such as the large amount of noise in the grinding environment, non-structural casting entities, and the inclination in the overall shape–time variation. Even in the face of complex technology and a variety of difficulties, modern casting grinding technology still demands large-batch production, low cost, fast response, thin brittleness, high precision, etc. The grinding process has a long history. This paper focus on its development from a human-operated, mechanical job, to an automatic grinding task based on compliant control theory. However, the methods mentioned above can no longer satisfy the current production need. In recent years, researchers have proposed intelligent grinding methods to meet the needs of modern casting production, and provided various strategies and alternatives to the challenges of machining accuracy, machining efficiency, and surface consistency. The research direction of casting polishing has mainly focused on online robot detection, material removal prediction, constant grinding contact force control, and high-precision matching. Although applications for online detection and constant grinding contact force control exist in industry, there are challenges in material removal prediction and three-dimensional high-precision matching. This paper also compares and analyzes the advantages and disadvantages of different grinding methods, and puts forward some research directions for future work, so as to promote more intelligent and efficient grinding of complex castings in practical application.

## 1. Complexity Description of Casting Grinding Process

At present, the casting grinding process is faced with many challenges, such as the large amount of noise in the grinding environment, non-structural casting entities, and the inclination in the overall shape variation, which limit the development of the casting grinding process. Therefore, the above problems need to be deeply analyzed.

### 1.1. Polishing Environment with Large Noise

**Figure 1:**
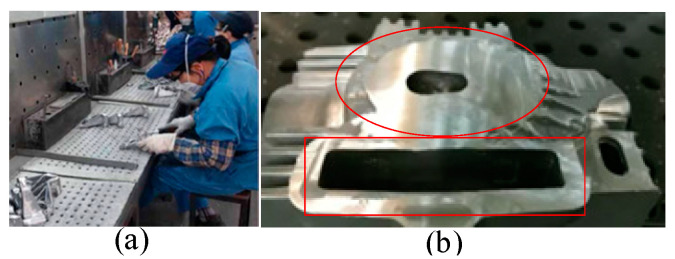
Casting post-processing site. (**a**) grinding scene (**b**) grinding effect (Based on practice, vibration can easily occur when grinding the red area of the cavity). During manual grinding, there is a long period of intense vibration exposure in the grinding process of hand-held workpieces, which causes arm vibration syndrome and harm to workers’ physical health [2]. Frequent vibration will also cause harm to the grinding workpiece and tools, because the tools and workpiece are vulnerable to damage during large vibrations. During mechanical grinding, a large holding force is needed to fix the casting workpiece, and this force may damage the casting part. A sensing device subject to large vibration interference cannot accurately capture the workpiece to be polished, and acquired data that contains a large amount of noise will affect the grinding accuracy. Large vibrations result in a large amount of noise for the control of the end-effector and have an impact on the stiffness of the equipment. Large vibrations also have a significant impact on the clamping of the workpiece, which means the workpiece can easily become loose. Large vibrations can also result in the splashing of hot debris.

Casting polishing is the main means of post-processing castings; however, dust generated in this process cannot be controlled, and large amounts of chromium dust and nickel particles will cause harm to the environment [1]. Manual grinding causes respiratory tract and lung diseases, and even pneumoconiosis, due to dust inhalation. Furthermore, artificial polishing efficiency is low, the consistency of workpiece products is poor, and the scrap rate is high. During mechanical grinding, high-density dust affects the equipment used in the clamping operation. High-density dust adheres to the equipment, and the dust reduces the accuracy and stability of the clamping equipment. When an intelligent grinding scheme is adopted, it is a challenge to seal robots and sensing devices, and thus prevent dust from entering. In addition, high-density dust blocks the surface of sensing devices, meaning they cannot accurately make judgements. At the same time, large vibrations have a serious impact on field operations, as shown in Figure 1.

**Figure 2:**
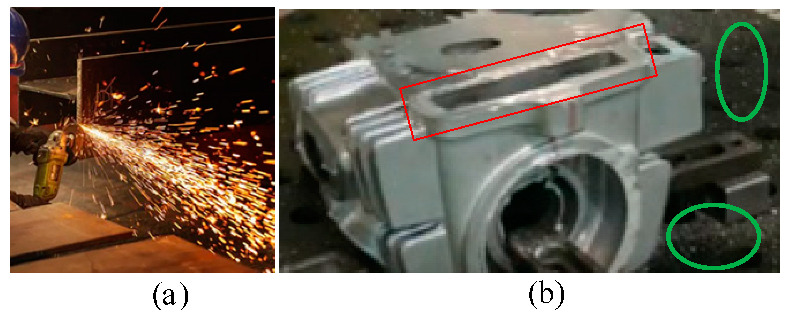
Debris splash scene. (**a**) grinding scene (**b**) grinding effect (thermal debris from sanding of the red part splashes on the green part).

In the process of grinding, the friction between the grinding head and the workpiece produces a large quantity of hot debris splashing, as shown in Figure 2. During manual grinding, a large quantity of thermal debris can block the sight of workers and seriously affect the accuracy of the workpiece. A large quantity of thermal debris having a high temperature can cause burns on the exposed skin of workers on site. A large quantity of uncontrolled high thermal debris can cause vision loss to the eyes of workers on site; debris accidentally flying into a human eye can seriously damage the lens and cause blindness [3]. Therefore, the grinding process is very precise and seriously affects the work efficiency. During mechanical grinding, the splashing of high-caloric debris will cause scalding on the surface of the nearby equipment, and an improper process can lead to the formation of chip tumors on the surface of the workpiece. High-caloric debris can also easily damage the outer layer of the power supply and the signal line. For the intelligent grinding system, high-caloric debris can interfere with the ability of the intelligent sensing equipment to obtain information. As a result, the obtained information may be incorrect and, therefore, not applied. This may lead to inaccurate pre-judgment and inaccurate planning strategies, causing sharp sound pulses at a minor level, or directly damaging the grinding equipment at a serious level.

The main cause of a sharp sound pulse is excessive grinding. In manual grinding, a sudden sharp sound pulse stimulates workers’ ears, which can damage hearing and cause hearing loss, and even cause perforation of the eardrum and deafness, resulting in a decline in the quality of work. During mechanical grinding, the sound can exceed the alarm of the equipment, which can result in damage to workers’ health and equipment during the operation of the equipment. For intelligent systems, due to the uncertainty in casting a deformation structure, the prediction may not be accurate, and a sudden sharp sound pulse can easily be produced. This can lead to damage to the equipment and the workpiece, and serious accidents can be caused.

There is an urgent need for industrial robots to realize unmanned casting post-processing because of the large amount of noise in the environment during casting post-processing. It is necessary to study and analyze the technology to overcome the challenges of high-density dust, large vibrations, splashing of high-temperature debris, and sharp sound pulses during the grinding process. In addition to the challenges during the grinding process, the non-structural characteristics in the solid design of castings and the time variation in the overall inclined shape during the casting process have a serious impact on the post-processing of castings.

### 1.2. Non-Structural Casting Entities

Castings usually have a three-dimensional surface structure, and there are a large number of non-structural concave surfaces and non-parametric convex surfaces. The concave surface cannot be easily polished inside by hand. Due to the large number of concave surfaces, during mechanical grinding it is difficult to find the datum surface or fix the combination surface that can be clamped. Furthermore, without a datum in machining, it is difficult to ensure accuracy. In addition, when an intelligent grinding method is adopted, data collection for concave surfaces having a large curvature can easily be blocked, resulting in incomplete data collection.

**Figure 3:**
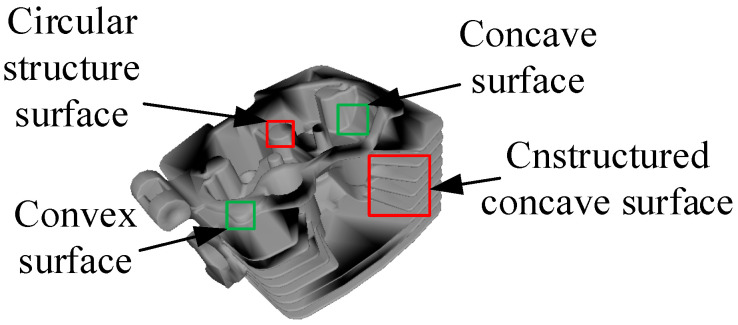
Unstructured cast solid surfaces.

As shown in Figure 3, the casting structure is complex and contains a large number of features. Among these, the simple features of the circular surface in the figure are not obvious, and there are a large number of non-structural surfaces [4]. In addition to casting the convex outside surface, there are a large number of internal concave surfaces, which are the most difficult structural concave surface. These surfaces reduce the measuring accuracy and the grinding accuracy [5]. The existing method uses artificial measuring and polishing, and the combination of mechanical and intelligent methods to conduct the processing is still in the experimental stage.

Due to the limitation of the non-structural curved surface casting process, the random interference of casting burrs, flanging, sagging, etc. not only seriously affects the determination of the datum, but also leads to a sharp change in the load during the process of grinding. Therefore, it is very important to accurately obtain 3D structure information of machined parts prior to grinding. The obtained 3D structure information can then be processed, and a planning grinding strategy can finally be adopted according to the actual situation. There will also be a time change in the overall shape of the casting tendency in the process of forming, which will increase the difficulty in the post-processing of the casting.

### 1.3. Tendency for the Overall Shape to Change over Time

**Figure 4:**
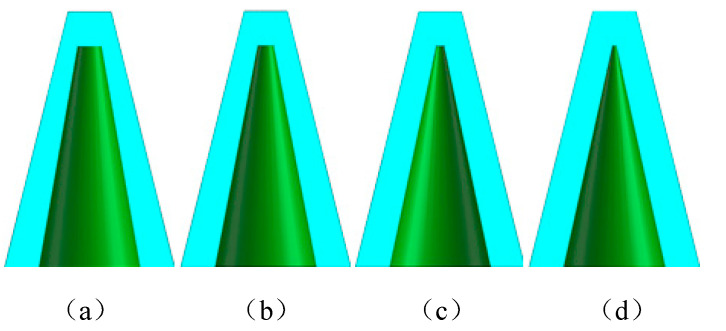
The change of the outer dimensions of the mold with the use of time (**a**) initial state (**b**) wear after a short period of use (**c**) wear after a longer period of use (**d**) failure state.

As the number of uses of sand molds and other molds increases, the bonding surface of the mold and casting will change regularly due to wear or adhesion, causing thickness change, tilt change, or overall scaling. In turn, this results in the loss of the original datum and the overall deformation of the casting, as shown in Figure 4. There are clear edges and corners in the (a) initial state mold cavity, and the deformation in (b) wear after a short use of time will appear with the increase in the number of uses. The deformation in (c) after a longer period of time and (d) failure status is too large and the shape in (a) is quite different. When the deformation is small, the casting will not be scrapped directly, but it will pose a challenge to the post-processing of the casting.

When manual grinding, to ensure the overall shape of the casting subject to tilt time change, workers need to judge whether deformation exists, and the form of deformation according to measurements, and then use a means of material removal for post-processing of the casting. The removal amount and removal method are determined according to the workers’ measurement and past experience. Because a certain deformation exists, excessive removal will directly lead to product scrapping. In mechanical grinding, it is also necessary to judge this situation according to the measured deformation. When removing materials for deformation correction, parameters of mechanical equipment need to be modified. However, repeated modification of the equipment parameters and the deformation judgment of castings will reduce the working efficiency and lead to product scrapping due to errors [6]. Intelligent methods can accurately match and identify tiny deformations and reach the accuracy of 0.052 mm [7]. However, judging and predicting the grinding mode based on experience, like that of grinding workers, is still in the experimental and verification stage.

Many challenges, such as the large amount of noise in the grinding environment, the time variation in non-structural casting entities, and the inclination in the overall shape, restrict the development of the casting grinding process. Non-structural castings and the overall tendency in the shape are problems in the casting design and production process, and a polishing environment subject to a large amount of noise is a problem in the post-processing of castings. It is necessary for researchers to improve the detection methods during casting and casting post-processing, and to use advanced industrial robots and sensors combined with advanced algorithms to replace human detection.

## 2. Development History of Grinding Process

### 2.1. Initial Manual Polishing

In the Stone Age, stone grinding [8] was mainly used to make various knives, stone axes, and other tools. In the Bronze Age [9], as the first country to employ copper smelting, China mastered the advanced casting post-processing technology. The rasp was used to compensate for casting defects, make the casting surface smooth, and make weapons and tools sharper and more polished [10]. After entering the Iron Age, rotary grinding tools appeared [11], which provided a reference for subsequent mechanical grinding. With the emergence of iron tools and rotary tools, and the advent of the first Industrial Revolution after the emergence of the steam engine, the manufacturing materials were mainly cast iron. Although casting products have changed, the grinding method still used manual grinding. Siemens developed the generator in 1866, providing technical support for mechanical grinding. In 1914, sandpaper developed by 3M in the United States resulted in a new grinding tool for post-processing for castings. Development subsequently entered a period of combining artificial and mechanical grinding in the post-processing of castings, which has continued until now.

To date, few studies have been conducted on manual grinding technology. In 2021, Zhao Jinghui et al. proposed and developed a hand-held rechargeable grinding tool that can assist personnel in maintaining power equipment subject to corrosion of metal parts and oxidation and heating of the contact surface, thus improving the grinding speed. In 2022, Whitmore, L et al. invented a precision grinding tool to be used for manual grinding of samples; the tool can be prepared by a 3D printer [12] and can grind samples with a surface accuracy of up to 10 µm.

Manual grinding is highly dependent on people, and its operating object is mainly small-batch samples. Manual grinding is far from satisfying the requirements of high-volume and low-cost workpiece grinding, and it is impossible to avoid the damage caused by noise, vibration, and scratches in the grinding process [13].

### 2.2. Mainstream Mechanical Grinding Process

**Figure 5:**
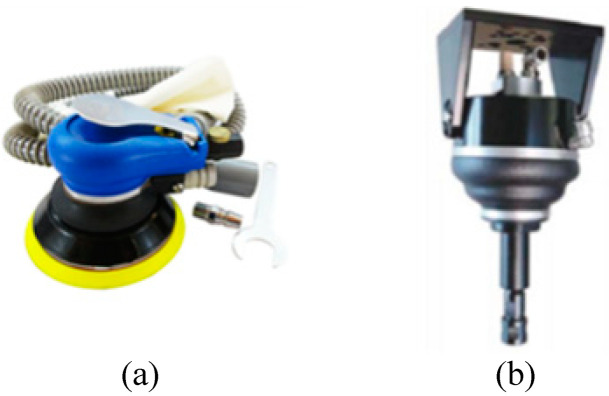
Pneumatic grinding equipment. (**a**) Pneumatic polishing machine. (**b**) Pneumatic deburring tool.

**Figure 6:**
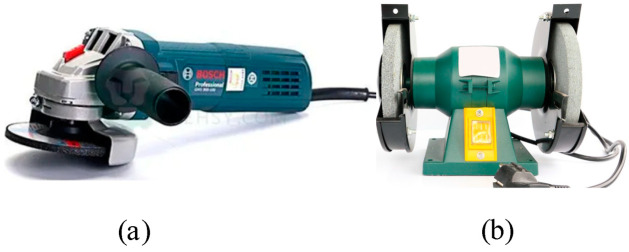
Electric grinding equipment. (**a**) Angled polishing machine. (**b**) Table-top grinding wheel machine.

As a result of the combination of the rotary grinding mode with a motor and pneumatic source, pneumatic grinding equipment (e.g., Figure 5) and electric grinding equipment (e.g., Figure 6) emerged. There are two ways of grinding with this equipment. In one, when grinding large parts, the workpiece to be polished is fixed, and the electric grinding equipment moves relative to the workpiece surface to complete grinding. In the other, when working on small parts, the electric grinding equipment is fixed, and the grinding is carried out by moving the workpiece to realize the relative movement of the rotating grinding head.

In 1875, Brown and Sheeper designed a sawing machine and universal grinder, and the grinding method combined with mechanical equipment began to appear. This grinding method, combining manual and equipment-based grinding, continues to this day. Special grinding machines have been designed for special parts according to the principle of the grinding machine [14]. In 1952, Massachusetts Institute of Technology successfully developed the world’s first Computer Numerical Control (CNC) milling machine [15]. The emergence of the CNC milling machine resulted in new mechanical grinding equipment and a new process for post-processing grinding of castings. When a CNC milling machine is used for casting post-processing, the workpiece to be polished is fixed to the standardized clamping device in the workspace of the milling machine, and the grinding tool is controlled by the CNC program for grinding processing [16]. Although the CNC milling machine can be used in the post-processing grinding of castings, its working space is small and the machine has poor flexibility. As a substitute for special machine tools, industrial robots are increasingly being used in the field of grinding. In 1986, Tate, A. R. of MIT used robots to realize automatic grinding of welds, and controlled the maximum normal force at 40 N and the maximum frequency of reference force at 2.3 Hz [17]. Later, another researcher, Peng J et al. [18], designed a passive grinding device, and studied the characteristics of the grinding process and the influence of the deflection angle in the passive grinding process. To meet the requirements of grinding complex parts, Guo Wanjin et al. from Harbin Institute of Technology designed and developed a compound 5-Dof working robot having large dexterity in its working space and dexterity in its attitude adjustment [19].

Table 1 scores the performance of different grinding methods. The average value (*Ep*) of the table is evaluated according to the favorable grinding degree (0–5), where 0 represents very poor and 5 represents very good. Weight *W*: Stiffness *W*1 = 0.6, flexibility *W*2 = 0.5, workspace *W*3 = 0.6, generality *W*4 = 0.7, cost *W*5 = 0.5.

The fuzzy mathematics weighted average evaluation value (*E_pw_*) and comprehensive evaluation value (*E_z_*) can be derived as follows:

(1) $$E_{pw}=E_{p}\times W$$

(2) $$E_{Z}=\sum_{i=1}^{5}\left(E_{pi}\times W_{i}\right)$$

The universal grinding machine has good performance in terms of rigidity and cost, but poor flexibility and a small working space, which are not suitable for the diversified needs of intelligent manufacturing. The CNC grinder has excellent performance in terms of stiffness and precision; however, for the processing of large complex surface parts, the cost of a high-precision CNC grinder is too high, and it is thus not suitable for procurement by small and medium-sized enterprises. In addition to its low stiffness, the robot grinding system has outstanding performance in terms of flexibility, workspace, versatility, and cost. The casting post-processing grinding process of an integrated robot grinding system has great development potential; however, the main structure design method used by grinding robots not only has a long design cycle, but also has poor grinding stability and stiffness, which is not suitable for product promotion. Therefore, the end-effector with compliant control theory combined with the grinding mode of industrial robots has been extensively studied by researchers.

### 2.3. Polishing Mode Based on Modern Control Theory

In the process of grinding, the change in the force is the key to improving the grinding performance to accurately control the force and displacement. It is difficult to control the contact force between the end-effector and the workpiece. Researchers have conducted a large number of studies on the control of grinding robot manpower, and found that there is a large interaction force between the end-effector and the workpiece during grinding, and that the control accuracy of this force will directly affect the machining accuracy [20,21,22,23] and the position control of the robot. Therefore, the simultaneous control of position and force is a challenge for grinding operations [24]. Researchers in the field of polishing robots use active compliance control, passive compliance control, and active and passive compliance control. The common means of active compliance control are power position hybrid control [25,26,27] and impedance control [28,29,30,31].

In 1993, Perdereau, V proposed a hybrid robot position control scheme. Subsequently, Zhou et al. proposed a hybrid control strategy of a grinding robot based on adaptive impedance control [32]. Tian, Y et al. [33] recently designed a fuzzy force controller that imitates human behavior in the process of rust removal. Subsequently, Zhao et al. developed a force/bit disturbance rejection control strategy based on fuzzy Proportion Integration Differentiation (PID). The proposed control strategy can achieve a force control accuracy of 13.4% for the expected 15 N contact force, and a material removal depth of 0.0362 mm can reach a precision of 1.2 μm [34]. Zhu et al. proposed a dynamic control method based on a one-dimensional force sensor PID controller. The roughness of the polished surface Ra < 0.4 μm, the material removal depth is more stable, the deviation remains at 0.003 mm, and the mean square deviation at 40 N is 0.37 N [32].

**Figure 7:**
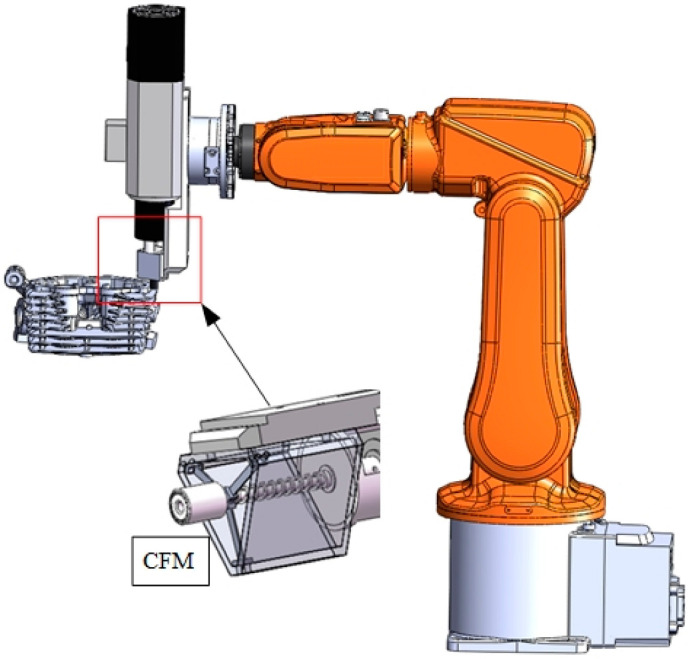
Autonomous sanding scenario using CFM end-effectors.

Grinding the workpiece to ensure accurate force control will reduce machining efficiency. The control algorithm is much more complex than the passive compliant constant-force mechanism. In addition, when the end-effector contacts the workpiece or the surface is irregular, force overshooting will be inevitable and relatively large. Therefore, researchers have used passive compliant mechanisms to develop end-effectors and conducted a large number of studies. Mohammad et al. proposed a forced-end-effector design, which was applied to the robot grinding system to make the grinding tool compliant and reduce the impact of vibration [35]. Y.M. Li proposed a constant-force mechanism (CFM) based on the combination of positive and negative stiffness mechanisms. The positive and negative stiffness of folded beam and bistable beam mechanisms was used to offset the zero stiffness to produce constant force, as shown in Figure 7. The proposed CFM can produce a stroke range of 2 mm in a constant-force mode [36], with a maximum of 12.63 N and a minimum of 12.43 N, and a flatness of 98.41%. Qingsong Xu and his team at the University of Macau proposed the design of an end-effector based on a constant-force mechanism for robot grinding. The designed industrial robot drives the end-effector to polish, and the end-effector passively adjusts the contact force. The precision of the force is ±0.3 N, resulting in high consistency of the workpiece’s surface quality [37].

From the development of grinding robots mentioned above, it can be clearly seen that the grinding robot is moving towards standardization. The control force and displacement accuracy are the main research directions of end-effector design. The use of constant-force grinding and constant-force clamping to precisely control the force greatly improve the grinding accuracy and clamping stability. However, due to the limitation of material properties and the size of the constant-force mechanism, the end-effector has insufficient load, an overly complex structure, and insufficient plane stiffness when the end-tool moves.

## 3. Demand Analysis of Grinding Technology for Modern Casting Production

As a result of the upgrading of industrialization and social progress, the market demand has increased for a casting grinding process having a large volume, low cost, fast response, high degree of precision, and thin brittleness.

### 3.1. Casting Scale Increase

**Figure 8:**
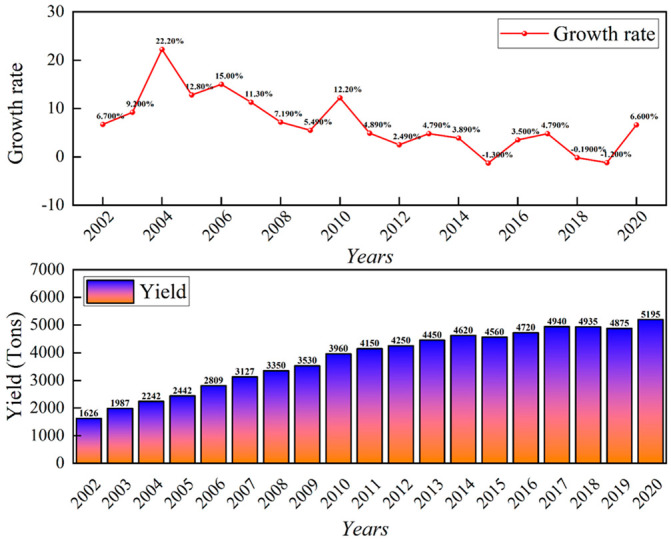
China’s casting production and growth rate in recent years.

China’s total foundry production reached 51.95 million tons in 2020 [38], an increase of 6.6% year-on-year, despite the impact of COVID-19. The casting output and growth rate in China in recent years and their changes are shown in Figure 8.

### 3.2. The Cost of Grinding Process Should Be Reduced

**Figure 9:**
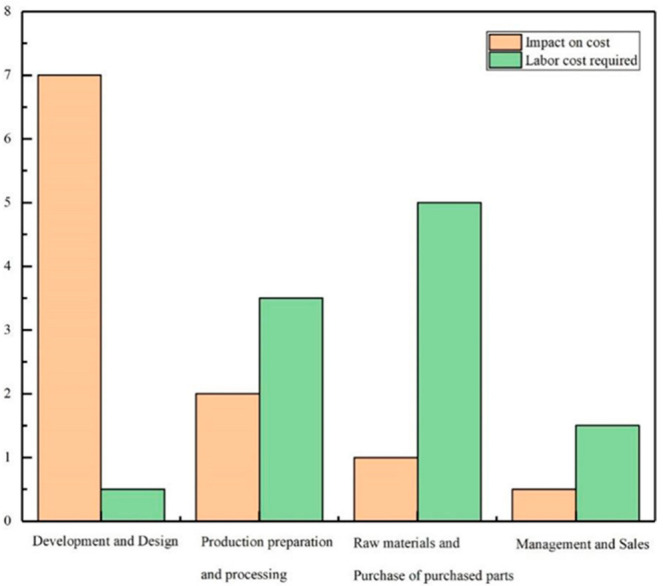
Cost impact.

In the market, it is necessary to reduce the cost of post-processing of large quantities of castings [39]. The cost can be roughly divided into four parts, as shown in Figure 9: development and design, production preparation and processing, procurement of raw materials and purchased parts, and management and sales [40]. The labor cost is not negligible in the process of grinding. Since the Industrial Revolution, the demand of enterprises for labor has been a topic of social concern, and technology also has an important impact on people and enterprises. As a result of the adjustment in the global industrial structure, especially in the situation of high-end competition in the manufacturing industry all over the world, boosting the technology dividend instead of the demographic dividend has become one of the main ways to reduce costs.

Upgrading the traditional grinding industry with modern equipment is the source of power and the necessary means of achieving a low cost. This is of great significance for promoting the progress of grinding technology, improving the quality of workers, improving the efficiency of casting enterprises, optimizing the industrial structure adjustment, and promoting the development of the manufacturing industry. At the same time, the market for a process having a fast response, high precision, and thin brittleness also has great expectations.

### 3.3. Technical Challenges of Complex Workpiece Grinding: Fast Response, Thin Brittleness, and High Precision

Fast response, high precision, and thin brittleness are further demands of the market on the basis of high volume and low cost, and they are also the aspects that researchers in the laboratory are tackling at present.

**Figure 10:**
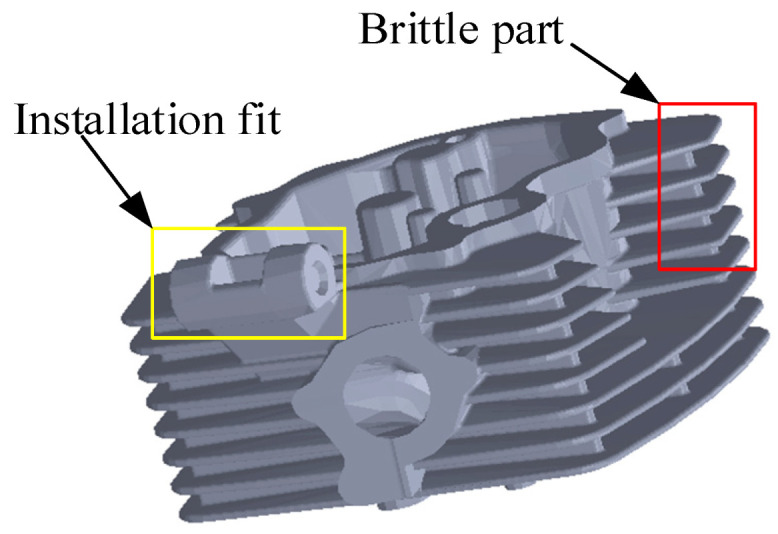
Complex casting.

The market requires low cost and mass post-processing of casting parts, and a fast manufacturing speed can greatly shorten product processing time in batch casting production and processing [37]. To achieve a high volume and low cost, a rapid pace of work is required. In turn, this requires a fast response. Ensuring a fast response is one of the problems that must be currently solved to improve the working speed. Without a fast response, it is very difficult to achieve mass production of polished products or low-cost production. Parts of the surface of the casting do not require high precision and can be sacrificed to improve speed. Where the casting is assembled with other parts (Figure 10), high precision is required. At the same time, parts generally processed by casting have a thin surface, which is a characteristic of casting parts with thin brittleness. However, due to the use of brittle materials and a complex surface, thin brittleness is more serious. At present, most grinding technology is aimed at the heavier parts, so thin brittle workpieces have resulted in new requirements for grinding technology. An accurate and fast response in force control technology, and a more accurate perception and planning strategy for grinding workpieces, are required.

In traditional high-precision grinding, market demand has not been met due to low efficiency, low accuracy, and damage to workers’ health. The market needs modern high-precision grinding technology. Modern grinding technology uses visual processing to achieve high-precision grinding; however, most of the grinding technology on the market uses 2D vision. In grinding system, it usually use a 2D visual method for grinding the workpiece, simple data relating to the workpiece can be easily acquired and processed. However, if the shape is complex, the acquired data will not be complete, and blocking, low precision, and path planning of the equipment will produce interference. The development of 3D vision has occurred very rapidly, and some researchers have started investigating the use of 3D vision in casting post-processing. However, problems of slow speed and low accuracy still exist because only high-precision algorithm processing can be used to obtain high-precision data; these data can then be used in the polishing and planning of high-precision grinding of workpieces. High-precision calibration and a registration algorithm are the necessary conditions for high-precision grinding. In recent years, in order to make up for the deficiency of traditional grinding, researchers have undertaken a large amount of exploration of intelligent grinding methods.

## 4. Classification of Intelligent Grinding Methods

In order to make up for the shortcomings of traditional grinding methods, and to im-prove the grinding efficiency and accuracy of castings, researchers have combined intelligent sensing devices and artificial intelligence algorithms in terms of judgment and prediction. The main achievements include grinding methods based on image vision, laser sensing, data-driven grinding prediction, 2.5D local feature information, and comparison between a design model and a 3D point cloud.

### 4.1. Judgment of Grinding Position Based on Image Vision

Manual grinding can be based on the actual situation, in which observation and measurement need to be made after the completion of a process, and the subsequent grinding strategy can be adjusted after real-time comparison of drawings and the size. With the extensive use of machine vision, humanoid real-time feedback and intelligent planning of the grinding strategy can be realized. With the support of artificial intelligence technology, vision sensors have been applied in a wide range of intelligent applications, which have played a positive role in inspiring the improvement in the grinding process [41]. Vision sensors have the advantages of non-contact detection, high precision, strong repeatability, fast speed, good stability, and low cost [42].

**Figure 11:**
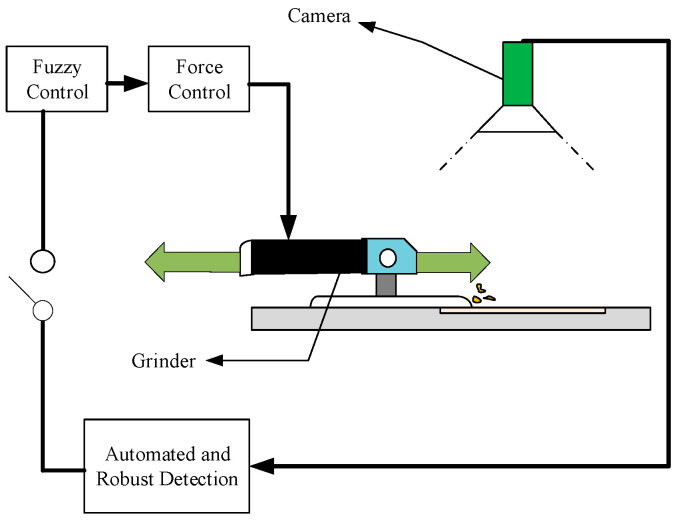
Visual servo sanding structure.

In 1981, Moravec proposed a corner detector for binocular vision image matching [43], and Harris proposed the Harris corner operator for image matching. In the early 21st century, a large number of correlation methods emerged. In 2001, a digital phase-shifting shadow technique was proposed, which takes only one image: the projection of the reference raster line on the surface of the deformed object. The phase shift is calculated by moving the virtual reference grating in its plane [44]. In 2010, Mohammadi proposed projecting the Moire grating on the surface of the object; the change in the shape of the object surface causes the phase change in the grating fringe, and the specific change in the phase can be extracted to obtain the three-dimensional in-formation of the object surface [45]. In recent years, there have also been many applications in industrial robots and grinding-related fields. Fang et al. proposed an active vision measurement framework for industrial robots, which is based on the motion planning technology of base sampling; the pictures taken by a camera at a certain frequency can be used for point cloud reconstruction of the measured workpiece [46]. Deng et al. developed an automated robotic repair system, which can complete defect detection and polishing within 3 min, and realize the surface burr removal process in the range of 60 cm × 60 cm and two elliptic ranges (the long axis is about 12 cm and the small axis is 7 cm) [47]. Tian, Y et al. [33] proposed a method to identify rust as a key technology in the process of rust removal. Rust detection is performed automatically by processing a series of camera images, and the visual servo control framework for the process of rust removal is shown in Figure 11.

In the process of grinding, the estimation of material removal and tracking of the grinding track directly affect the grinding accuracy. At present, in order to obtain the actual material removal amount, the main method uses offline or online measurement to establish a mathematical prediction model. In recent years, researchers have conducted a large of amount research on the estimation of material removal using image vision. Joshi et al. used a machine vision method to acquire and recognize texture features of surface images on polished surfaces, and used a regression model based on machine vision parameters to evaluate surface roughness [48]. Wang et al. used a two-dimensional convolutional neural network learning algorithm to monitor the material removal method, and extracted the features of color, texture, and shape from visual signals. These features constitute a two-dimensional feature matrix as the input parameter, and the material removal rate in the belt grinding process as the output parameter. This method can be used to predict the material removal rate of different sand belt specifications and different grinding parameters [49], and is suitable for the regression prediction of the material removal rate under typical working conditions.

Li et al. studied the mechanical spark generation of seven metals, namely, Q235 steel, 304 stainless steel, TC4 titanium alloy, 6061 aluminum alloy, H62 bronze alloy, AMAK3 zinc alloy, and AZ31B magnesium alloy. The relationship between the physical and chemical properties of friction sparks and their generation was evaluated. For 6061 aluminum alloy, H62 bronze alloy, AMAK3 zinc alloy, and AZ31B magnesium alloy, no bright friction sparks were observed at the maximum friction velocity of 12 m/s and the maximum surface pressure of 3.75 N/mm^2^, due to low hardness, high thermal conductivity, low melting point, and lack of carbon [50].

The results show that the estimation of material removal and tracking of the grinding track directly affect the grinding accuracy in the grinding process. Only the method based on machine vision can produce obvious friction sparks for metals having higher hardness, because soft metals cannot be predicted by the obvious external picture phenomenon; hence, there are limitations. The data-driven approach of multi-information fusion provides another approach for prediction with more complete information. The judgment grinding based on visual sensing can be used for the grinding work of industrial structures, but its robustness is affected to a certain extent due to the complex grinding environment and insufficient environmental illumination.

### 4.2. Judgment of Grinding Position Based on Laser Sensing

In the process of casting grinding, uncertain and strong disturbances, such as temperature, noise, vibration, dust, and light, are inevitable, and limit the popularization and use of vision sensors. Laser sensors can make up for some of the above deficiencies, especially those caused by dust [51].

**Figure 12:**
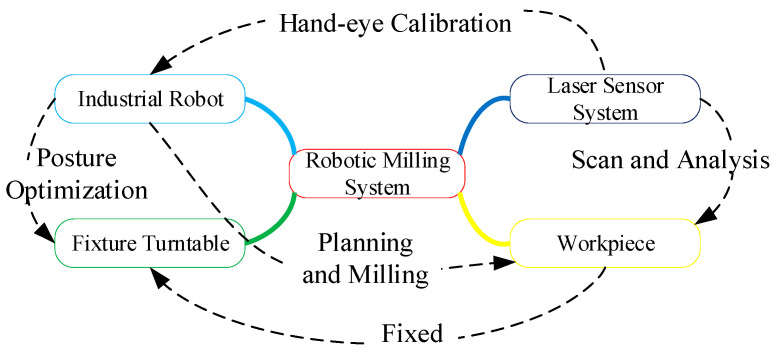
Robotic milling process system.

As early as in the 1970s, Nitzan et al. used the distance and intensity information of the laser ranging system to describe indoor scenes, and the stability and reliability of laser ranging were fully verified [52]. Laser scanning technology has been further developed in the field of surveying and mapping. In the 1980s and 1990s, Kak proposed the installation of a monocular laser vision sensor at the end of a robot to scan the surface of the object being measured. Lindstrand studied the laser measurement method for measuring the diameters of pipes and bars in the steel industry. Early applications were characterized by low accuracy, slow processing speed, and vulnerability to environmental interference. In recent years, researchers have studied the industrial applications of laser sensors. Xu Xiaohu from Huazhong University of Science and Technology used laser sensors to optimize the traditional hand–eye calibration algorithm and all its aspects [53,54,55,56,57], and established a hand–eye calibration model based on the tool center coordinates to accurately obtain the spatial pose relationship between the robot and the laser scanner, as shown in Figure 12. The fitting error was calculated as F = 0.060 mm. More importantly, the entire automatic calibration process lasts only 20 s when a proper path is planned, which greatly saves calibration time. A. Seidel tried to use a cooperative laser profilometer to obtain geometric shapes at the clamping position, and used an adaptive milling path planning method to automatically offset changes in the position and shape of parts caused by accidents [58].

**Figure 13:**
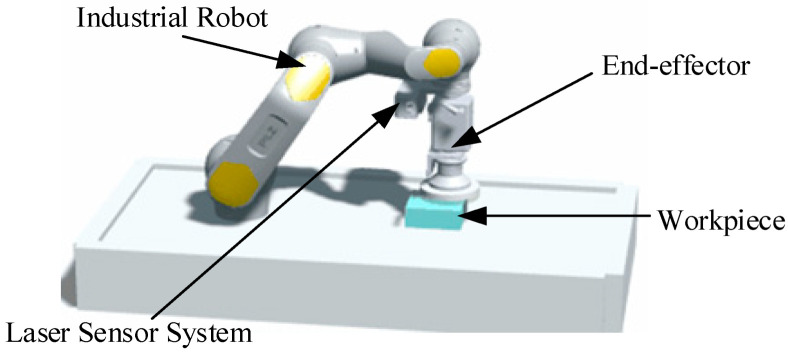
Robot laser scanning and grinding method.

Laser sensors have also been widely used in the field of grinding. Gao, Y et al. carried out research on robot grinding technology for welding pretreatment technology of large parts [59], and adopted a laser profilometer for on-site measurement, planning and processing. At AUTOMATICA 2018 in Munich, Fraunhofer IPA and PILZ, a member of the Ros Industrial Consortium, presented an on-site measure–plan–process grinding robot, as shown in Figure 13. On the basis of previous research, Ge et al. further proposed and constructed a robot welding-line grinding system based on laser sensors [60]. A sensing device and a self-made grinding tool were integrated at the end of the robot for the grinding operation. After rough grinding, the weld height was kept at about 0.1 mm, and the average surface roughness after fine grinding was 0.351 μm.

The polishing robot based on a laser sensor has a unique advantage in the face of the harsh grinding environment in a factory, i.e., it is not affected by the environmental light, and the precision can also meet the requirements for the post-processing of casting parts. The grinding accuracy can be predicted online by a data-driven prediction method.

### 4.3. Abrasive Quantity Prediction Based on Data-Driven

Grinding surface roughness is considered to be one of the key indicators of machining quality [61]. However, due to the randomness and complexity of abrasive particle distribution, it is difficult to predict. In order to accurately estimate the roughness of the polished surface, it is necessary to obtain the amount of material removed by grinding in many ways. The amount of material removed by grinding is related to multiple parameters [62], such as feed speed, rotational speed, contact stress, grinding time, workpiece material, geometric characteristics of the workpiece, and condition of the grinding head. In order to obtain the desired amount of removed material, the above parameters should be optimized and combined [63].

#### 4.3.1. Model-Based Material Prediction

The most widely used model-based method is the Preston equation, according to which the material removal rate during grinding is proportional to the pressure and the relative velocity between the medium and the workpiece. However, due to the lack of a method to measure relative speed and pressure, this empirical law has not been applied to finishing [64]. Preston’s law is expressed as:

(3) $$\frac{dV}{dt}=\frac{1}{k}F*v$$

where dV/dt is the surface indent distance (mm/s), *t* is time, *V* is indicates volume. 1/*k* is the unit of Preston coefficient *k*, *F* is the contact force, v is the relative speed between the workpiece and the tool. [65].

Equation (3) was studied by researchers, and Castillo-Mejia proposed a correlation expression of the local material removal rate in grinding based on the Preston equation. Lee et al. [66] established a model of the grinding material removal rate based on the Preston equation for independently changing pressure and speed in real time. In the above study, the Preston coefficient, grinding force, and rotational speed were regarded as constant values. During the grinding process, the grinding head grinds the workpiece, and the material properties of the workpiece surface change with the accumulation of heat. Therefore, material removal cannot be accurately predicted using a constant polishing coefficient, K. Pan R et al. [67] proposed extraordinary k-constructed correction functions based on interface friction coefficients and verified their validity by experiments. In actual grinding, the tool and workpiece do not have a single point of contact, but have regional contact, which is inconsistent with Preston’s hypothesis. Calculations using the classical Preston hypothesis will lead to inaccurate estimates of material removal [68]. Therefore, based on Hertz contact theory and a local area grinding model, Wang et al. further predicted the cutting depth of robot belt grinding [69] and found that when the cutting depth was about 0.3 mm, the prediction error was less than 3.1%. Compared with the simplified Hertz theory model, the root mean square value and mean absolute percentage error of the material removal model proposed by Zhu et al. were reduced from 2.401 to 1.725 and 18.426 to 14.942%, respectively, considering the elastic deformation of contact. As a result of the widespread use of artificial intelligence, many methods based on data-driven material removal prediction have been proposed to solve the material removal prediction problem.

#### 4.3.2. Data-Driven Prediction of Material Removal

Due to the complexity of the grinding process, some of its parameters cannot be accurately detected in real time, which limits the application of model-based methods in engineering implementation. An increasing number of researchers are using data-driven methods to predict material removal.

As early as 2005, the data-driven method was applied to predict material removal. Panda D predicted the material removal rate using an artificial feedforward neural network [70]. Mathew, J. et al. used an artificial neural network to analyze the material removal amount and established a parameter optimization model [71]. Wang et al. proposed a material removal prediction algorithm using neural networks and genetic algorithms [72]. In order to ensure the whole process of grinding work detection, it is necessary to build a real-time monitoring welding seam clearance prediction system. Thus, David Jin Hong studied a deep learning vision system to automatically detect the grinding end-points of welds in the grinding process and monitor the geometric changes in the grinding welds. The deep learning method, which conducts end-to-end processing on a large number of experimental grinding data, can obtain good material removal prediction results [73]. In order to improve the accuracy of the polishing robot, Zhang et al. combined acoustic sensing and the XGBoost algorithm to predict the material removal in sand belt polishing [74], with an average absolute percentage error of 4.373%.

When predicting material removal in grinding, the model-based method mainly focuses on the basic parameters in the grinding process; thus, the model-based method yields high-precision material removal results. However, many factors affect the prediction of grinding materials. The data-driven method represented by neural networks provides a solution for processing and analysis.

### 4.4. Grinding Method Based on 2.5D Local Feature Information

Two-dimensional image information is concentrated on the plane, and the depth information provided by robot grinding is not accurate. Therefore, more accurate 2.5D information has the advantage of representing 3D objects, thus improving the chance of reliable recognition [75].

In 2008, Verma, A proposed a 2.5D processing feature recognition system. It was used to screen out 2.5D part features to determine the machining direction [76]. In 2009, Siebert et al. extended the 2D SIFT algorithm to 2.5D for application [77]; the proposed algorithm can be directly matched using local features of 3D rotation invariance. Zhang Yuwei et al. proposed a method to reconstruct the underlying 3D shape from a 2.5D bas-relief, and optimized the face shape through normal transfer and Poisson surface reconstruction [78]. Zhang et al. constructed a 2.5D height field for portrait relief to enhance the appearance of the portrait [79], and the 2.5D technology was applied to the processing of the portrait relief. The technology can also be applied to the surface grinding of a portrait relief.

**Figure 14:**
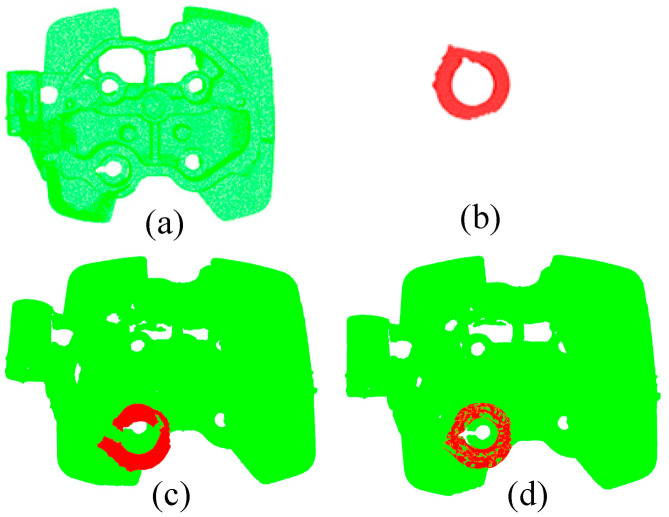
Local feature based matchingMatching of local features. (**a**) Workpiece data template (**b**) Local feature data template (**c**) Coarse alignment positioning (**d**) Fine alignment positioning.

Figure 14a shows the data template of a workpiece to be polished obtained by a sensing device; Figure 14b shows the local template features of the workpiece to be polished; Figure 14c shows the approximate location of B in a rough registration; and Figure 14d shows the precise location of B in A through fine registration. After registration, it is convenient for grinding tools to plan the machining route of the workpiece, which can greatly improve the machining accuracy. High-precision matching is essential for automatic grinding.

The above grinding method based on 2.5D local feature information has low accuracy in the depth direction, and can be used for machining parts with low accuracy requirements. The disadvantage of using local information polishing is that additional steps are required to derive the surface information, and, like the 2D method, this requires representation from a separate specific viewpoint. There are errors in other parameters calculated by the geometric reasoning method, and the feature recognition system based on a prompt requires prompt features in the workpiece. Therefore, 3D vision technology must be used for deep and accurate machining.

### 4.5. Grinding Method Based on Comparison between Design Model and 3D Point Cloud

**Figure 15:**
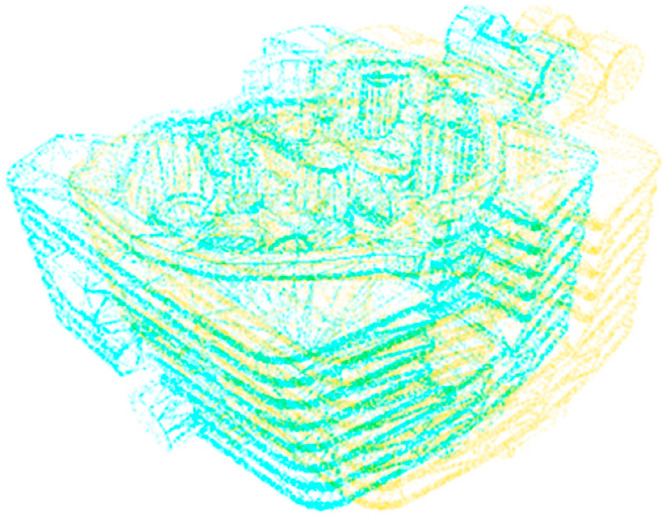
Comparing the matching graph.

In the grinding method based on the comparison between the design model and the 3D point cloud, the 3D design model of the workpiece to be polished is subtracted from the real-time model of the detected workpiece [80], and the difference between the real-time model and the original 3D digital model represents the part to be polished [81], as shown in Figure 15. Two sets of data are matched to facilitate the comparison of workpiece defect errors. Kuss, A proposed a method to detect the workpiece shape deviation to adapt to the robot grinding process. The method uses the model of product design to design dimensional tolerance specifications to predict possible changes in the workpiece geometric model, using the Iterative Closest Point (ICP) method to match each point cloud with the measurement point cloud from the workpiece [80]. In order to further improve efficiency and accuracy, Wei proposed a method to automatically evaluate the machining allowance of casting parts. The scanned point cloud data were aligned with the design model through two stages of “initial alignment” and “best registration” to find the best registration and evaluate the machining allowance based on the registration results [82].

In terms of workpiece grinding, Hu et al. developed a robot deburring and chamfering system [83], in which the human operator can select any feature on the Computer Aided Design (CAD) model and export the selected feature for the tool path of trajectory generation. However, artificial feature selection was inefficient. Zhang et al. proposed an adaptive grinding method for precision casting of blades with geometric deviation [5]. The measured data of blades were matched with the design model, and the corresponding matching matrix was solved to determine the position of the cast blades. In order to further improve the accuracy of automatic laser deburring of ceramic cores, Huang et al. proposed a point cloud registration method combining global and local feature information [84], and the final overall error was less than 35 µm.

**Figure 16:**
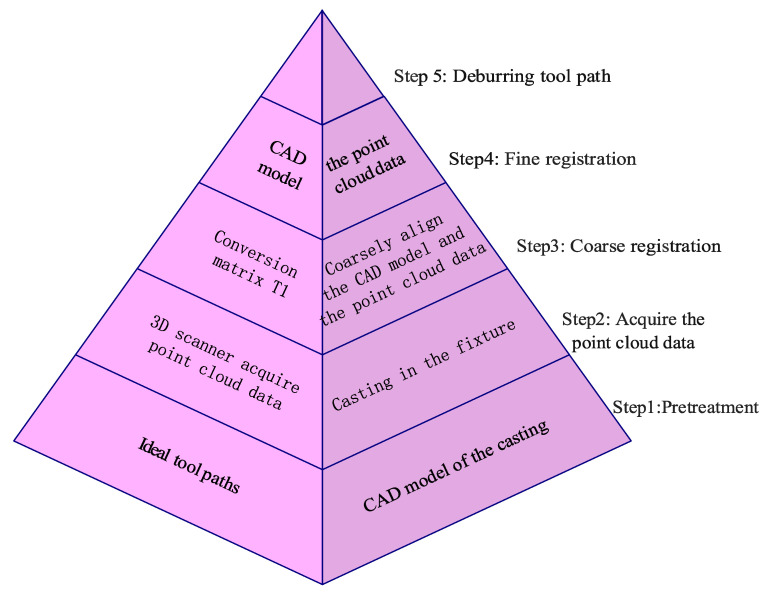
Key steps in grinding path generation.

Due to the uneven deformation of casting parts, the optimal machining route is unknown. Therefore, the ideal grinding machining route can be used as a benchmark to quantitatively measure the accuracy of different grinding paths, so as to determine which registration method can grind paths with high precision. The key steps of grinding path generation are shown in Figure 16.

The method based on the comparison of a design model and 3D point cloud data has become an effective detection method for many digital design processes. Point cloud matching is divided into two stages: rough matching and fine matching. Rough matching algorithms include principal component analysis, four-point congruency, three-dimensional normal distribution transformation, and local feature description, such as Fast Point Feature Histogram Feature. The precision of rough matching cannot reach the precision specified in the manufacturing industry, so the precision must be further improved through fine matching.

The traditional fine matching algorithm is represented by the ICP algorithm. The principle of the algorithm is that the rotation matrix *R* and translation vector *T* are obtained by solving Formula (4) of the ICP algorithm for point set *P_i_* and *X_i_*.

(4) $$\sum_{}^{2}=\sum_{i=1}^{N}||X_{i}-(R•P_{i}+T)|{|}^{2}=\min$$

The algorithm has a good registration effect for point clouds having a high overlap rate and close initial position, but has shortcomings in terms of the amount of computation required and the iterative convergence speed. With rough matching, the ICP algorithm can solve the problems of a low degree of overlap and large difference in initial position. As a result of the development of artificial intelligence algorithms, researchers have also carried out a large amount of research on the intelligent registration of the point cloud based on deep learning.

(A) Traditional point cloud registration method

**Figure 17:**
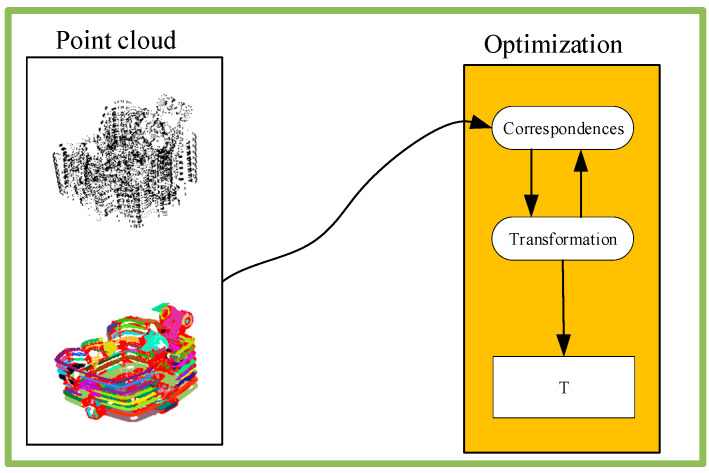
Optimization-based registration method.

Based on the traditional registration algorithm, the speed and accuracy of the algorithm are further improved through analysis; the performance principle of the traditional algorithm is shown in Figure 17 [85].

As early as 1992 Besl, P. J used a general ICP algorithm for accurate and efficient registration of 3D shapes. An important application scenario of this algorithm is to register the data repeatedly measured by the sensing device of rigid objects with the ideal geometric model before shape inspection, so as to improve the accuracy of the sensing data [86]. The main disadvantages of ICP are slow convergence [87], sensitivity to outliers, missing data, and partial overlap. In 2013, Pauly M proposed Sparse ICP, which achieved robustness at the cost of computational speed through Sparse optimization [88]. In 2021, Zhang proposed a fast convergence robust registration method. It was proved that the classical point-to-point ICP can be regarded as a domination–minimization (MM) algorithm, and an Anderson acceleration method was proposed to accelerate its convergence [89]. In addition, the Welsch function-based robust error measurement method [90] was also introduced to achieve accuracy that was similar to or better than that of Sparse ICP, while improving speed by an order of magnitude. The fast convergent robust ICP registration method provides theoretical and method support for the acquisition of perception data of the grinding robot, and can further improve the complexity of the grinding workpiece.

(B) Intelligent point cloud registration method

**Figure 18:**
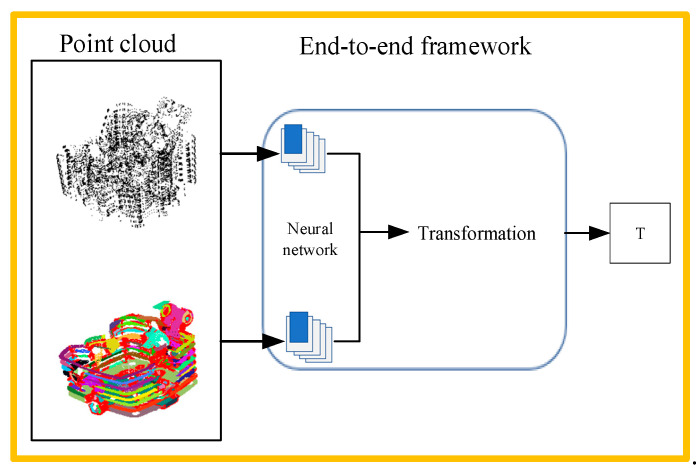
Learning-based point cloud registration algorithm.

The method based on end-to-end learning can be used to solve the registration problem using an end-to-end neural network, as shown in Figure 18. The end-to-end learning method transforms the registration problem into a regression problem, and the transformation estimation is embedded in the neural network.

Due to the late development of intelligent point cloud registration methods, Qi proposed a neural network named PointNet in 2017, which provides a unified framework for applications such as classification, segmentation, and scene semantic analysis. According to this framework, Yasuhiro Aoki proposed expanding the PointNet and LK algorithms into a single trainable recursive deep neural network in 2019, opening up a new exploration path for the application of deep learning in point cloud registration [91]. In 2020, Yuewang He proposed a registration method combining PointNet++ and ICP. PointNet++ can extract multiple features that are used as a basis for registration, using ICP algorithms to calculate rotation and translation. Recently, some researchers proposed solutions for intelligent registration. Liu et al. proposed a robust point cloud registration method based on deep learning [92] called Point cloud Deep Cyclic Net, which uses the adjustment network based on principal component analysis to quickly adjust the initial position between two pieces of the point cloud. Perez proposed a rigid point cloud registration method called Point Cloud Registration Learning (PREL) [93], which allows the registration of point clouds with high displacement or occlusion. The PREL algorithm does not need an iterative process and estimates point distribution in a non-parametric way. In the highly occluded point set, the ICP method showed an average root mean square error (RMSE) of 98.8, followed by a Deep Closest Point of 32.51 and PREL of 0.75.

The study of relevant literature in the field of grinding indicates that the efficiency of the three-dimensional neural network algorithm is lower than that of the traditional registration algorithm. To date, although the intelligent registration algorithm has good performance in terms of accuracy, the calculation time is long and the cost is high, whereas the traditional algorithm is fast and efficient. As a result of the continuous progress in intelligent technology and the updating of algorithms, intelligent registration algorithms have more potential than the traditional registration algorithm.

The design model is used to compare the workpiece to be polished. As a result, some defects to be polished are detected and the situation of the polished workpiece is obtained in real time by the feedback of the sensing device. Starting from the design source, the errors generated in other processes in the casting post-processing and polishing process are reduced.

## 5. Summary and Prediction

**Figure 19:**
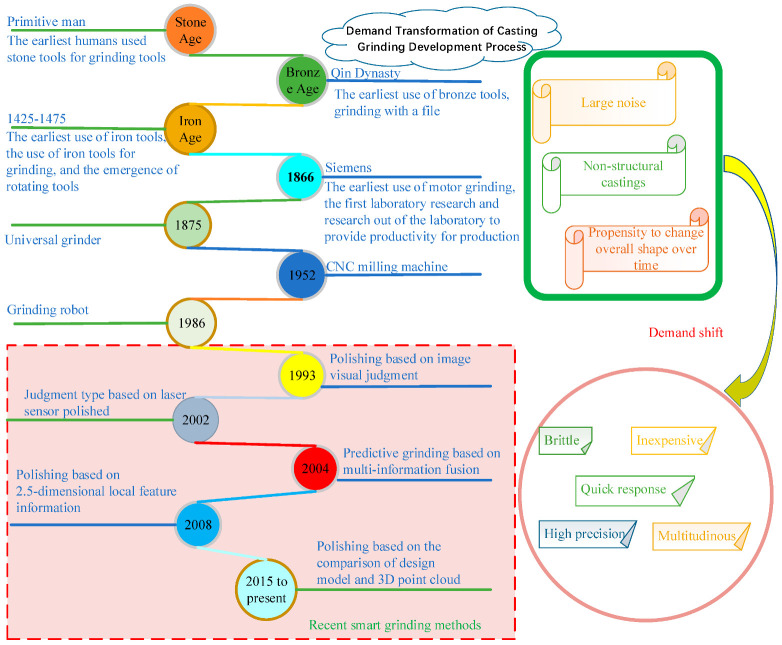
Demand conversion and grinding process development.

The previous section can be summarized as shown in Figure 19. As demand changes and modern technology advances, grinding methods are being developed.

Different polishing methods have different advantages, but they also have disadvantages, as shown in Table 2.

According to Table 2, the manual approach can be used to process almost any casting grinding according to manual experience, which is flexible. However, with the increase in casting output, manual grinding efficiency is low, and can seriously harm the lungs and arms of grinding workers. In contrast to manual grinding, mechanical grinding does not require workers to contact the workpiece, and the system has good rigidity and small vibration, so the grinding accuracy is high. However, due to its poor flexibility and small space, only specific castings can be polished. In view of the small workspace and poor flexibility, industrial robot grinding based on the compliant control theory adopts force and position control for grinding. In this approach, grinding precision is high, and the workspace is large and flexible, but rigidity is poor. In recent years, many intelligent grinding methods have been developed. Image vision-based grinding position judgment uses visual perception equipment and visual judgment algorithms, so that the grinding equipment performs the judgment function; however, this approach is seriously affected by the environment and is limited to the two-dimensional plane. The laser sensing-based polishing part judgment method uses a laser sensing device and a judgment algorithm; this ensures the polishing device has the function of accurate judgment and provides good robustness, but the data acquisition speed of the device is slow. The data-driven grinding quantity prediction method uses advanced sensing equipment combined with a visual prediction algorithm to predict the final grinding effect using the image and force data in the grinding process; however, the obtained image data are seriously affected by the material and environment. The grinding method based on 2.5D local feature information adopts the combination of a feature recognition algorithm and the depth pre-estimation method on the basis of the sensing device to achieve grinding to a depth based on part of the depth information; however, the depth information is not accurate and the feature recognition needs to be set several times. Based on the design model and the 3D point cloud contrast grinding method, a laser sensor and registration algorithm can be used to obtain 3D point cloud data with 3D information, which can provide accurate information for path planning.

Table 1 shows that industrial robots have advantages in processing large parts. Compared with CNC machine tools, the mechanical arm performs well in terms of cost, and has large space and good flexibility, but the consistency of feeding is poor. It is difficult to realize automation of robot processing of large or lightweight workpieces, and the series structure has low stiffness, resulting in poor stability of the processing process. Therefore, the parallel grinding robot has great development potential.

In the process of visual recognition, the grinding environment is complex, and sensing equipment having good adaptability and high accuracy is the key breakthrough direction. Sensing equipment needs to accurately perceive the position and shape of the workpiece and other information. After sensing, a high-precision matching visual algorithm can be used. There is urgent demand for improvement in registration.

The feedback control of the constant grinding contact force is required in high-speed grinding systems. High-precision control of the grinding force is very important for the consistency of the grinding surface of complex parts. The application of the constant-force mechanism in the grinding field provides a new research idea for accurate control of the grinding force.

In the process of grinding, the amount of material removed directly affects the grinding accuracy. In order to obtain an accurate estimate of the amount of material removed, offline measurement is needed to establish a prediction model. At present, the prediction model has low accuracy and produces a serious environmental impact. A model that can accurately predict material removal can obtain higher efficiency and precision in the process of material removal by grinding.

## Figures and Tables

**Table 1:** Comparison of conventional grinding machines with robotic grinding systems.

	Universal Grinding Machine	Special Grinding Machine	Numerical Control Grinding Machine	Robot Grinding System
**Stiffness**	4	4	4	1
**Flexibility**	2	0	3	5
**Workspace**	1	1	2	5
**Versatility**	2	0	3	5
**Cost**	3	2	2	4
**Synthesis** **Evaluation value**	6.3	4.1	8.2	11.6

**Table 2:** Comparison of grinding methods.

Grinding Method	Core Technology	Advantage	Shortcoming	Literature
Manual grinding	Human experience	Flexible	Inefficient and damaging to the body	[8,9,10,11,12]
Mechanical grinding	Machine tool equipment	High precision	Poor flexibility and small space	[14,15,16,17,19,94]
Grinding based on compliant control theory	Force and position control	High precision and large working space	Poor rigidity	[18,32,34,35,36,37]
Judgmental grinding based on visual sensing	Visual perception devices, visual judgment algorithms	Intelligence has a judgment function	Limited two-dimensional plane space judgment, affected by the environment	[33,46,47]
Judgmental grinding based on laser sensing	Laser perception equipment, judgment algorithm	The equipment is robust and accurate	The device acquires data slowly	[45,58,59,60]
Predictive grinding based on machine vision	Visual prediction algorithms, advanced perception devices	Material removal prediction, intelligent material removal	Only harder materials have better predictions and are greatly disturbed by the environment	[48,49]
Predictive grinding based on multi-information fusion	Predict the model, training data	Material removal prediction	Low prediction accuracy and a large amount of training data are required	[64,65,95,96]
Grinding based on 2.5D local feature information	Depth pre-estimation method, feature recognition algorithm	Has depth information	The depth information is inaccurate and the feature recognition needs to be set multiple times	[76,78,97]
Based on the design model and the 3D point cloud comparison grinding method	Laser sensors, registration algorithms	Accurate route planning with 3D information	The amount of information is large, and the amount of calculation of the intelligent algorithm is large	[5,84]

## Data Availability

Not applicable.
